# The stability of FKBP9 maintained by BiP is crucial for glioma progression

**DOI:** 10.1016/j.gendis.2023.101123

**Published:** 2023-09-22

**Authors:** Shirong Li, Wangxiao Xia, Bin Sun, Weiyan Peng, Dong Yang, Jing Gao, Shuai He, Hua Yang, Yongjie Zhu, Hu Zhou, Tingxiu Xiang, Qingpeng Kong, Xudong Zhao

**Affiliations:** a Division of Abdominal Tumor Multimodality Treatment and Laboratory of Animal Tumor Models, Cancer Center and State Key Laboratory of Respiratory Health and Multimorbidity and Frontiers Science Center for Disease-related Molecular Network, West China Hospital, Sichuan University, Chengdu, Sichuan 610041, China; b State Key Laboratory of Genetic Resources and Evolution/Key Laboratory of Healthy Aging Research of Yunnan Province, Kunming Institute of Zoology, Chinese Academy of Sciences, Kunming, Yunnan 650223, China; c Key Laboratory of Molecular Oncology and Epigenetics, The First Affiliated Hospital of Chongqing Medical University, Chongqing 400016, China; d Department of Analytical Chemistry and CAS Key Laboratory of Receptor Research, Shanghai Institute of Materia Medica, Chinese Academy of Sciences, Shanghai 201203, China; e The Third People's Hospital of Yunnan Province, Kunming, Yunnan 650600, China

**Keywords:** BiP, Endoplasmic reticulum stress, FKBP9, Glioma, Knockout mice

## Abstract

FK506-binding protein 9 (FKBP9) is involved in tumor malignancy by resistance to endoplasmic reticulum (ER) stress, and the up-regulation of FKBP9 is associated with patients' poor prognosis. The current knowledge of the molecular mechanisms is still limited. One previous study showed that FKBP9 could confer glioblastoma cell resistance to ER stress through ASK1-p38 signaling. However, the upstream regulatory mechanism of FKBP9 expression is still indistinct. In this study, we identified the FKBP9 binding proteins using co-immunoprecipitation followed by mass spectrometry. Results showed that FKBP9 interacted with the binding immunoglobulin protein (BiP). BiP bound directly to FKBP9 with high affinity. BiP prolonged the half-life of the FKBP9 protein and stabilized the FKBP9 protein. BiP and FKBP9 protein levels were positively correlated in patients with glioma, and patients with high expression of BiP and FKBP9 showed a worse prognosis. Further studies showed that FKBP9 knockout in genetically engineered mice inhibited intracranial glioblastoma formation and prolonged survival by decreasing cellular proliferation and ER stress-induced CHOP-related apoptosis. Moreover, normal cells may depend less on FKBP9, as shown by the absence of apoptosis upon FKBP9 knockdown in a non-transformed human cell line and overall normal development in homozygous knockout mice. These findings suggest an important role of BiP-regulated FKBP9-associated signaling in glioma progression and the BiP–FKBP9 axis may be a potential therapeutic target for glioma.

## Introduction

FKBPs are members of the immunophilin family and are being identified as new targets for tumor therapy.^1^, ^2^, ^3^, ^4^ They contribute to diverse cellular processes, including cellular signal transduction, transcriptional regulation, protein transport, and protein folding. FKBPs contain peptidylprolyl cis–trans isomerase (PPIase) domains, and most of them are also followed by tetratricopeptide repeat domains or carboxy-terminal EF-hand domains.^5^^,^^6^ FKBP12 containing one PPIase domain binds to oncoprotein MDM2 and induces MDM2 degradation to enhance the sensitivity of chemotherapy in cancer.^7^ FKBP members containing the tetratricopeptide repeat domain are co-chaperones of Hsp90 and represent potential therapeutic targets for cancer.^8^ FKBP4 containing tetratricopeptide repeat domains has been reported to regulate the stability of estrogen receptors in breast cancer^9^ and integrate FKBP4/Hsp90/IKK with the FKBP4/Hsp70/RelA complex to promote lung cancer progression.^10^ FKBP10, the protein most structurally similar to FKBP9, has been reported to interact with ribosomes to regulate protein translation in sustaining lung cancer growth.^11^

FKBP9 harbors four PPIases and two carboxy-terminal EF-hand domains. Its structure suggests that it participates in important physiological processes related to protein function.^12^ The PPIases catalyze the conversion of proline from trans form to cis form in residues. As a rate-limiting enzyme in many protein folding reactions, PPIases have the potential to assist protein folding and regulate protein functions.^13^ Additionally, the EF-hand domain binds to Ca^2+^ and is involved in calcium-mediated protein recycling mechanisms.^12^ Notably, FKBP9 is associated with cancers, including distant metastasis and the promotion of cancer progression. It correlates with distant metastasis and serves as an independent prognostic marker in prostate cancer.^14^ Some studies have also shown that FKBP9 is up-regulated in colorectal cancer^15^ and FKBP9 mutation is related to breast cancers.^16^ Mounting evidence confirms that FKBP9 relates to brain tumors.^17^ FKBP9 is a poor prognostic biomarker for isocitrate dehydrogenase 1/2 (IDH) wild-type glioblastoma.^18^ FKBP9 up-regulation predicts poor survival of patients with glioma.^19^^,^^20^ Another study further confirmed that FKBP9 up-regulation could confer glioblastoma cell resistance to endoplasmic reticulum (ER) stress through ASK1-p38 signaling.^21^ However, the upstream regulatory mechanism of FKBP9 expression in glioma remains unknown and is worth exploring.

In this study, we try to identify factors that regulate FKBP9 expression in glioma and illustrate the impact of FKBP9 ablation on the overall survival of genetically engineered FKBP9 conditional knockout mice with orthotopic glioma. Our results provide insight into the development of novel potential therapy strategies for glioma.

## Materials and methods

### Cell culture

HCT116, SMMC7721, HepG2, SKOV3, HeLa, A549, H1299, MCF-7, MDA-MB-231, T98G, U87-MG, U251, and HEK-293T cells were cultured in Dulbecco's modified Eagle's medium (Gibco, USA) supplemented with 10% fetal bovine serum (Millipore, USA) and 1% penicillin‒streptomycin (Life Technologies, USA) in 5% CO_2_ at 37 °C. Cell lines were authenticated using short tandem repeat profiling.

### Plasmid construction and lentiviral packaging

FKBP9 shRNA sequences were cloned into pLKO.1-puro or tet-pLKO-puro vectors. The target sequences were shFKB9P-1#: 5′-CGCACGTTTGACACGTACATT-3′ and shFKBP9-3#: 5′-CGAGAGACGTTTCGTGAAGAT-3′. FKBP9 cDNA was purchased from YouBio Inc, China. BiP cDNA was cloned from T98G cells and verified by sequencing. Flag-HA-labeled FKBP9, BiP, and BiP-mutant (synonymous mutations (from cgcattgatactagaaat to cgGattgaCactagGaaC) within the shRNA target region) were cloned into the pTomo vector modified with a puromycin selection marker, respectively. BiP lentiviral preparation was performed as described previously.^22^ The silencing efficiency of FKBP9 was quantified with Western blotting and quantitative PCR using FKBP9-specific primers (FKBP9-F: 5′-GAAAAGCGAAGGATTGTGGTCCC-3′; FKBP9-R: 5′-TGCTGATGGAGTCCGAAGGGTT-3′).

### EdU (5-ethynyl-2′-deoxyuridine) incorporation for proliferation assays

Cells were incubated with 10 μM EdU for 1 h, followed by fixation with 4% paraformaldehyde and permeabilization with 0.5% Triton X-100. EdU staining was performed according to the manufacturer's protocol (Invitrogen, USA), and cell nuclei were stained with DAPI (4,6-diamidino-2-phenylinodole) (Sigma, USA). Images were captured with a fluorescence microscope and EdU-positive cells were quantified.

### Immunofluorescence staining

Cells cultured on glass coverslips were fixed with 4% paraformaldehyde, permeabilized with 0.3% Triton X-100, blocked with 10% goat serum, and incubated with the following primary antibodies: FKBP9 (Life Technology, USA), Cleaved-caspase3 (Cell Signaling Technology, USA), HA-tag (Cell Signaling Technology, USA), and Flag-tag (Life Technology, USA). After washing with 0.25% Tween-20, the cells were incubated with CY3 or FITC-labeled secondary antibodies (Life Technology, USA), followed by staining with DAPI.

### Western blotting

Cell pellets were lysed in RIPA buffer (Beyotime, China) containing protease and phosphatase inhibitors (Thermo Scientific, USA) on ice. Lysates were centrifuged at 16,000 *g* for 30 min, and the supernatant was quantified using the bicinchoninic acid (BCA) Protein Assay Kit (Beyotime, China). Equal quantities of denatured protein were fractionated by SDS‒PAGE and transferred onto nitrocellulose membranes. The membranes were then blocked in 5% bovine serum albumin for 1 h and incubated overnight with FKBP9 (Proteintech Group, USA), BiP, HSF1, Calnexin, Hsp90, Hsp70, Hsp60, Hsp40, CHOP, HA-tag (Cell Signaling Technology, USA), or β-actin (Thermo Scientific, USA) primary antibodies at 4 °C. The membranes were then washed and incubated with the appropriate secondary horseradish peroxidase-conjugated antibodies. The blots were analyzed using an ECL reagent (Millipore, USA).

### Co-immunoprecipitation and immunoprecipitation

Cells expressing EGFP-HA, FKBP9-HA, and BiP-HA protein were lysed in co-immunoprecipitation buffer (50 mM Tris–HCl, pH 7.4, 150 mM NaCl, 3 mM EDTA, 0.5% Triton X-100, 1% PMSF, and 1% protease and phosphatase inhibitor cocktails) and rotated at 4 °C. The lysates were then centrifuged at 8200 *g* at 4 °C for 10 min. The supernatant was co-immunoprecipitated using HA (Thermo Scientific, USA) or FKBP9 (Thermo Scientific, USA) antibody. The co-immunoprecipitated proteins were boiled and resolved via SDS‒PAGE, followed by silver staining analysis. Protein identification was performed using liquid chromatography–tandem mass spectrometry on a Q-Exactive mass spectrometer (Thermo Scientific, USA). For ubiquitination assay, T98G cells expressing FKBP9-HA were infected with shBiP-3# and then treated with 5 μM MG-132 for 6 h. The cell lysates were immunoprecipitated using an HA antibody, and ubiquitinated FKBP9 was detected by immunoblotting with a ubiquitin antibody (HuaBio, China).

### Surface plasmon resonance (SPR) assay

SPR assays were performed using a BScreen LB 991 Label-free Microarray System (BERTHOLD TECHNOLOGIES, Germany) according to the manufacturer's instructions. The sensor chips were provided by Betterways Inc., China. Proteins were printed on the biosensor chip using a BioDot™ AD-1520 Array Printer (BIODOT Inc., USA) and covalently attached to the chip surface using a UV Spectroirradiator 1020 (Amersham Life Science, USA) to trigger the photo-cross-linking reaction. The human recombinant proteins BiP (TP305859, Origene, USA), FKBP9 (TP322307, Origene, USA), and β-actin protein (ab240844, Abcam, USA) were diluted in running buffer (containing 10 mM HEPES, pH 7.0, 3 mM EDTA, 150 mM NaCl, and 0.005% (v/v) P20 surfactant) at concentrations of 200, 400, 800, 1600, and 3200 nM for affinity analysis. The protein sample was injected at 5 μL/min for 10 min in the associating stage, followed by the injection of running buffer at 5 μL/min for 6 min in the dissociating stage. At the end of each associating–dissociating cycle, the sensor chip was regenerated using 10 mM glycine-HCl (pH 2.5) at 20 μL/min for 30 s. The signals generated during these steps were collected and the output was depicted as affinity curves, and the association constant (K_a_) and dissociation constant (K_d_) were automatically calculated by means of slope fitting. These SPR assays were performed by Betterways Inc. (Guangzhou, China).

### Immunofluorescence and immunohistochemistry

Tissues were fixed in 4% paraformaldehyde and embedded in paraffin. Five-micrometer sections were stained at 4 °C overnight with the following primary antibodies: FKBP9 (Life Technology, USA), Ki-67 (Vector, USA), cleaved-caspase3 (Cell Signaling Technology, USA), and CHOP (Cell Signaling Technology, USA). This was followed by incubation with fluorescence-labeled secondary antibodies and counterstaining with DAPI. The microarrays were stained with the primary antibodies FKBP9 (Life Technology, USA) and BiP (Life Technology, USA), followed by incubation with horseradish peroxidase-conjugated secondary antibodies and 3,3′-diaminobenzidine. Tissue microarrays were purchased from Shanghai Chengke Biotechnology Co., Ltd.

### Generation of Fkbp9 knockout mice

Fkbp9 conditional knockout mice were generated at Shanghai Biomodel Organism Science & Technology Development Co., Ltd. Targeted embryonic stem cells were verified through genomic PCR and sequencing. Male chimeras were bred with B6;SJL-Tg(ACTFLPe)9205Dym/J^23^ female mice to delete the PGKneo cassette and generate F1 progeny. Fkbp9+/LoxP (Fkbp9^+/L^) and FKBP9^+/L^ were crossed to obtain homozygous Fkbp9^L/L^ mice. Constitutive knockout mice were obtained by mating Fkbp9^L/L^ mice with Vasa-cre transgenic mice.^24^

### Intracranial tumor models

Intracranial models were generated as previously described.^25^ Ras-V12-IRES-Cre-ER-shp53 lentivirus was injected into the hippocampus of 8-week-old Fkbp9 conditional knockout (Fkbp9^L/L^) mice using a stereotaxic instrument. One week later, tamoxifen in corn oil was administered at 4 mg/20 g body weight using a feeding needle every 3 days. The mice were observed for survival, and the Kaplan–Meier survival curve was drawn using the GraphPad Prism statistical software.

### Statistical analysis

Samples with RNA-seq for FKBP9 and BiP expression of patients with glioma and normal tissues were from TCGA and GTEx datasets. Samples with RNA-seq and survival data were from the Chinese Glioma Genome Atlas (CGCA), and the survival rate was estimated by the Kaplan–Meier method and log-rank test. Pearson correlation test was performed to assess the expression pattern between FKBP9 and BiP. Pearson correlation coefficient *R* ≥ 0.3 (or ≤ −0.3) was considered a strong positive (or negative) correlation. *P* < 0.05 was considered statistically significant.

## Results

### BiP interacts with FKBP9 in glioma cells

**Figure 1:**
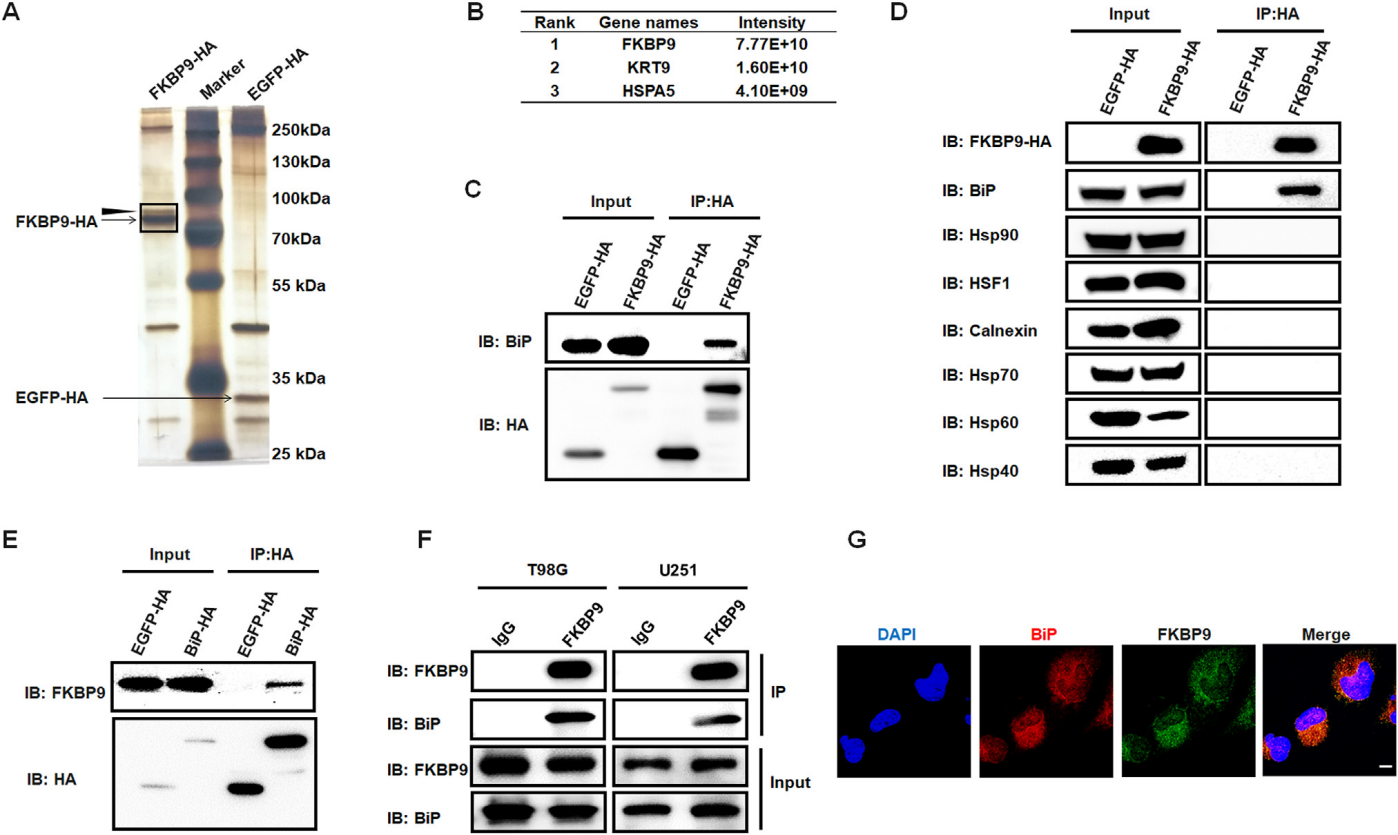
BiP interacts with FKBP9 in glioma cells. **(A)** Co-immunoprecipitation assay was performed using HA-tag antibody in T98G cells infected with FKBP9-HA or EGFP-HA lentivirus and subjected to silver staining. EGFP-HA served as a control vector. The bands in the black frame were identified with LC‒MS/MS. **(B)** Top 3 proteins identified in the gel bands (A) via LC‒M S/MS assay. The order is in descending order of protein intensity. HSPA5 (BiP) was identified as the most abundant chaperone. **(C)** Proteins were extracted from T98G cells expressing FKBP9-HA or EGFP-HA, subjected to co-immunoprecipitation assay using the HA-tag antibody, and detected by Western blot using HA-tag and BiP antibody. **(D)** The interaction between FKBP9 and chaperones (BiP, Hsp90, HSF1, Calnexin, Hsp70, Hsp60, and Hsp40) was detected by co-immunoprecipitation assays using HA-tag antibody in T98G cells expressing FKBP9-HA or EGFP-HA. **(E)** Proteins were extracted from T98G cells expressing BiP-HA or EGFP-HA, subjected to co-immunoprecipitation assay using the HA-tag antibody, and detected by Western blot. **(F)** Co-immunoprecipitation assays were performed to assess the endogenous interaction between FKBP9 and BiP using FKBP9 antibody in protein lysate of T98G and U251 cells. IgG served as a negative control. **(G)** Double immunofluorescence staining in T98G cells with FKBP9 and BiP antibodies. Scale bar = 10 μm.

**Figure S1:**
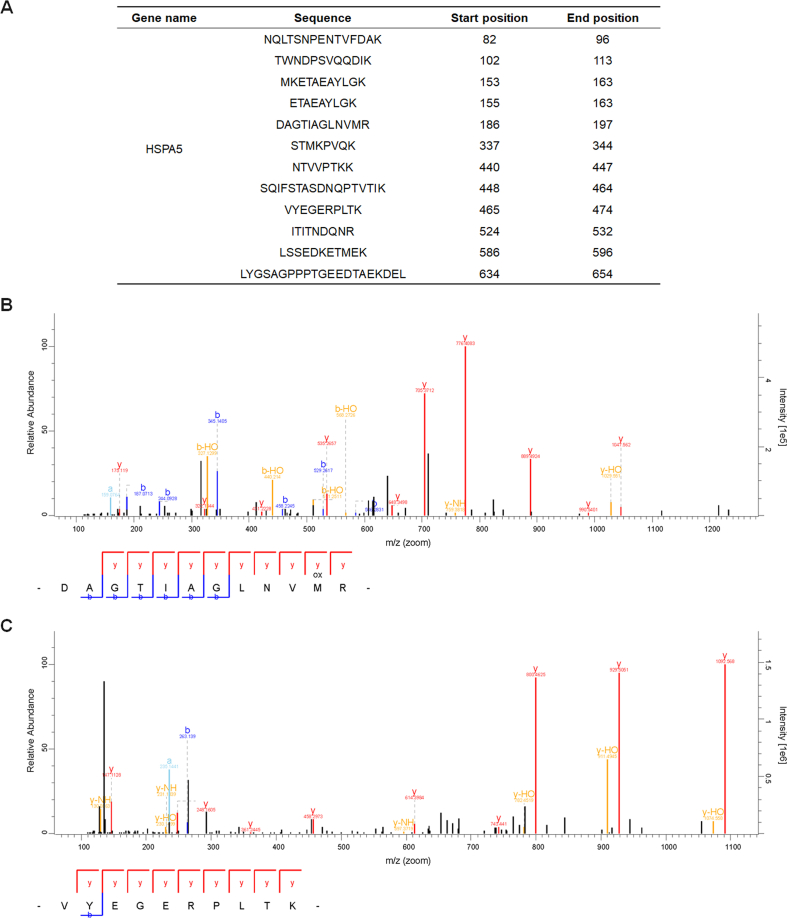
The peptides of HSPA5 identified by LC‒MS/MS. **(A)** The identified 12 peptides of HSPA5 in the black frame (Fig. 1A) by LC‒MS/MS. **(B, C)** The representative product ion spectra in (A). The product ion spectra of HSPA5 peptides of 186DAGTIAGLNVMR197 (B) and 465VYEGERPLTK474 (C).

**Figure S2:**
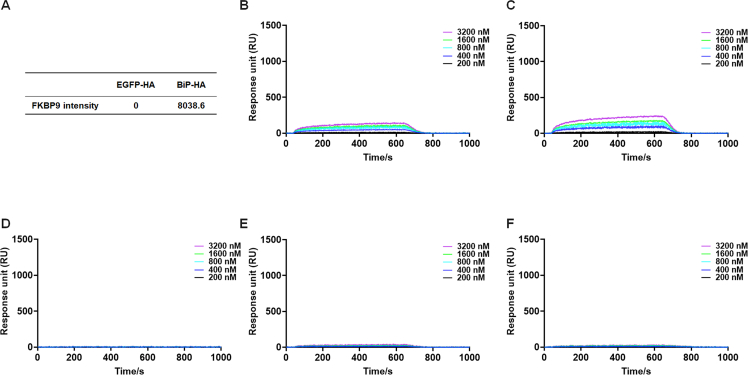
BiP interacts with FKBP9. **(A)** Proteins were extracted from T98G cells expressing BiP-HA or EGFP-HA and subjected to co-immunoprecipitation assay using the HA-tag antibody. The elution of co-immunoprecipitation assay was identified by LC‒MS/MS. EGFP-HA served as the control. The numbers in the table represent the intensity of the FKBP9 protein identified by LC‒MS/MS. **(B–F)** Sensorgrams of the interactions of β-actin with different concentrations (200 nM, 400 nM, 800 nM, 1600 nM, and 3200 nM) of FKBP9 (B) and BiP (C), the interactions of PBS with different concentrations (200 nM, 400 nM, 800 nM, 1600 nM, and 3200 nM) of FKBP9 (D) and BiP (E), and the interactions of PBS with different concentrations (200 nM, 400 nM, 800 nM, 1600 nM, and 3200 nM) of β-actin (F).

To explore the regulatory factors of FKBP9 expression, we first investigated the proteins that may bind to FKBP9. T98G cells were infected with HA-tagged EGFP or FKBP9 lentivirus and the cellular lysates were co-immunoprecipitated with HA antibodies. The precipitated proteins were analyzed by SDS‒PAGE and silver staining. Silver staining detected a unique protein band (arrowhead-indicated) in proteins immunoprecipitated by FKBP9 but not in the EGFP control group (Fig. 1A). The bands in the black frame were identified with LC‒MS/MS. It has been reported that some members of the FKBP family are co-chaperones, and the co-chaperones typically cooperate with chaperones.^26^^,^^27^ Among the identified proteins, HSPA5 (BiP) was the most abundant chaperone (Fig. 1B; Fig. S1), and BiP has been reported to play crucial roles in glioma.^28^ Western blot also confirmed the existence of BiP in the complex of FKBP9 (Fig. 1C); however, no other chaperones involved in protein folding, such as Hsp90, HSF1, calnexin, Hsp70, Hsp60, and Hsp40, were confirmed (Fig. 1D); and vice versa, BiP-binding proteins were co-immunoprecipitated with the HA antibody in T98G cells infected with HA-tagged-BiP lentivirus. FKBP9 was identified only in the BiP group and not in the control (EGFP) group (Fig. S2A). Western blot analysis confirmed the existence of FKBP9 in the co-immunoprecipitation products of BiP but not EGFP (Fig. 1E). Furthermore, co-immunoprecipitation also confirmed the interaction between endogenous FKBP9 and endogenous BiP in T98G and U251 glioma cells (Fig. 1F). In addition, confocal immunofluorescence microscopy showed that endogenous FKBP9 co-localized with BiP in T98G cells (Fig. 1G).

### BiP binds directly to FKBP9 with high affinity

**Figure 2:**
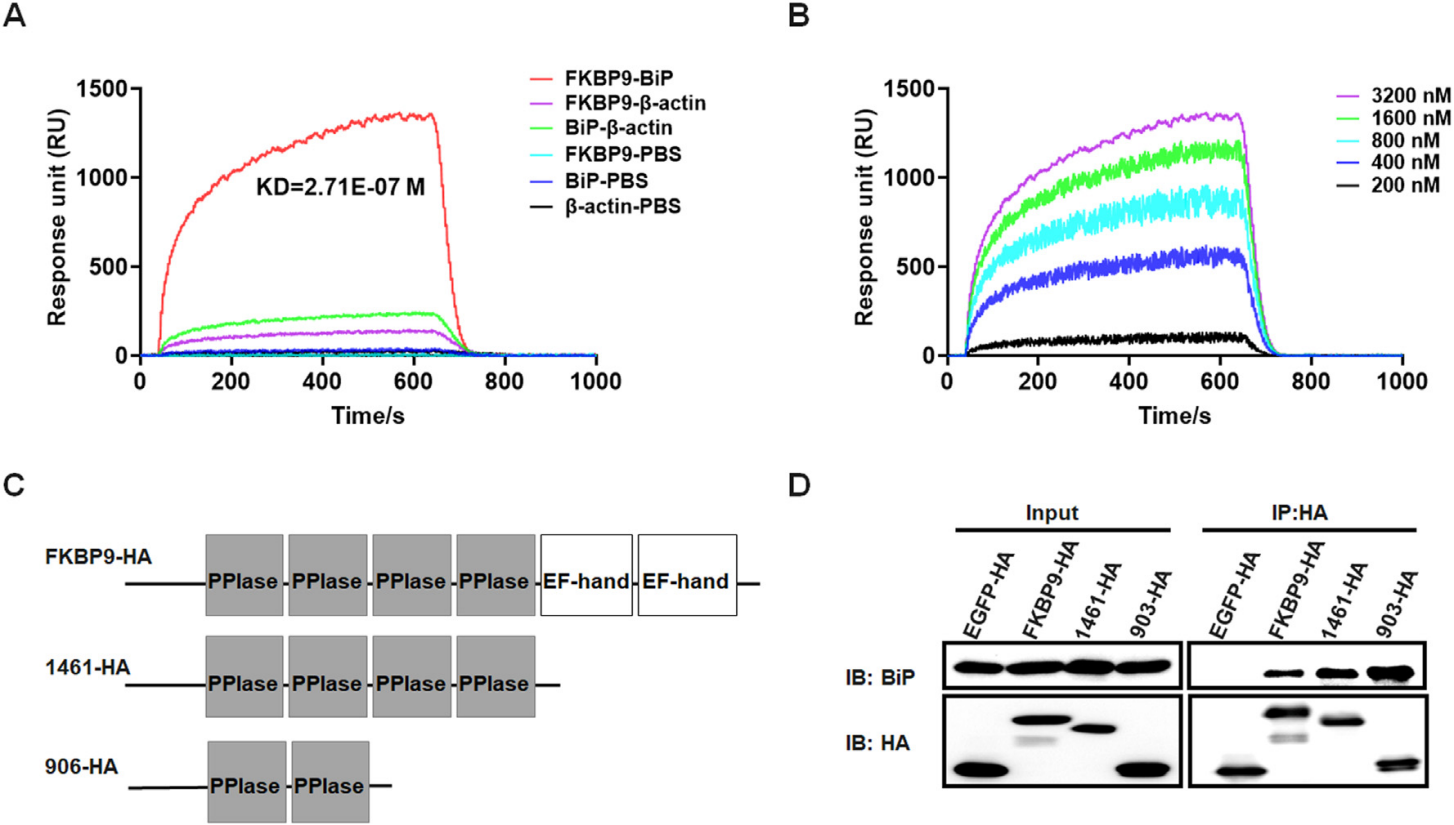
BiP directly binds to FKBP9 with high affinity. **(A)** Biophysical analysis of the interaction between FKBP9 and BiP by surface plasmon resonance assays. The red line represents the interaction between FKBP9 and BiP, and the Kd value is 2.71E-07 M. β-Actin and PBS were used as the control and background noise control, respectively. **(B)** Binding curve of the interaction between different concentrations of FKBP9 and BiP. The purple, green, sky blue, dark blue, and black lines represent different concentrations of FKBP9 (200 nM, 400 nM, 800 nM, 1600 nM, and 3200 nM, respectively). **(C)** Schematic diagram of FKBP9 truncations used in the co-immunoprecipitation assay in (D). 1461-HA represents the HA-tagged FKBP9 truncation without two EF-hand domains, and 903-HA represents the HA-tagged FKBP9 truncation without two EF-hand domains and two C-terminal PPIase domains. **(D)** The interaction between FKBP9 truncations and BiP by co-immunoprecipitation assays using HA-tag antibody in T98G cells expressing FKBP9-HA,1461-HA, 903-HA, or EGFP-HA.

To clarify whether BiP directly interacts with FKBP9, we subjected purified FKBP9 and BiP to SPR analysis. The low Kd value (2.71E-07 M) for the interaction of FKBP9 with BiP indicates a strong interaction between FKBP9 and BiP (Fig. 2A). β-Actin and PBS, which were used as controls, showed weak or no interaction with FKBP9 and BiP. Furthermore, SPR analysis of BiP and different concentrations of FKBP9 showed that the interaction was dose-dependent (Fig. 2B), while that for β-actin and PBS was not (Fig. S2B-F). These results demonstrate that FKBP9 directly binds to BiP with high affinity.

FKBP9 harbors four PPIases and two EF-hand domains. To identify the region responsible for BiP binding, lentiviral plasmids expressing the different truncated HA-tagged FKBP9 fragments were constructed (Fig. 2C). HA-tagged EGFP lentiviral plasmids were used as the negative control. Cells infected with these lentiviral particles were harvested for co-immunoprecipitation with the HA antibody. While no BiP was detected in the negative control via Western blotting, the full-length FKBP9 as well as the truncated FKBP9 containing PPIase bound to BiP (Fig. 2D), indicating that the PPIase domain is sufficient for FKBP9 interaction with BiP.

### BiP increases the stability of FKBP9

**Figure 3:**
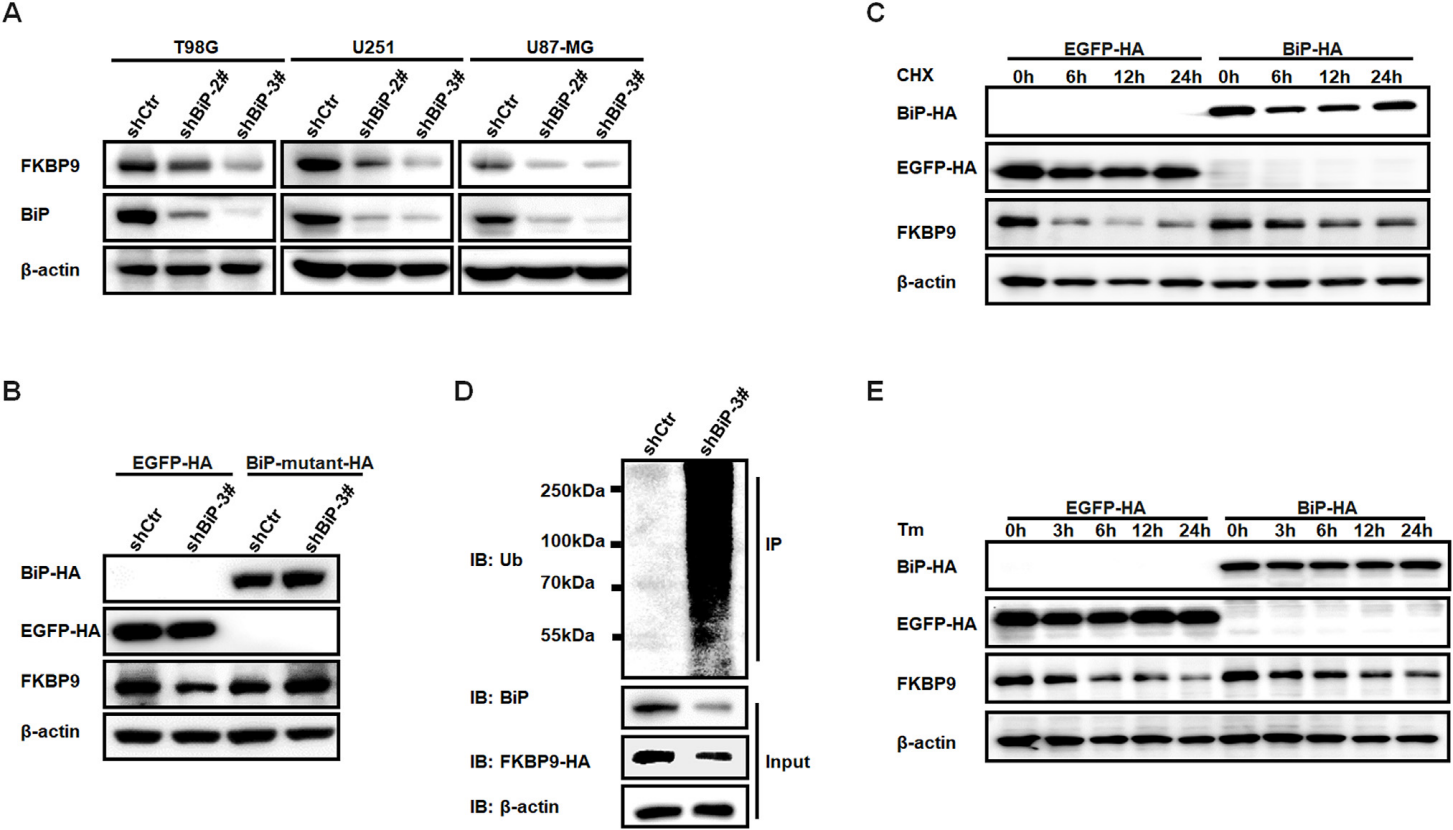
BiP increases the stability of FKBP9. **(A)** Western blot analysis of the protein level of FKBP9 upon BiP knockdown by shRNAs of shBiP-2# and shBiP-3# in T98G, U251, and U87-MG cells. shCtr was used as the control shRNA. β-Actin was used as the loading control. **(B)** T98G cells expressing HA-tagged BiP-mutant and EGFP were infected with shBiP-3# lentivirus and then lysed to detect FKBP9 expression using Western blotting. **(C)** Western blot analysis of FKBP9 protein in BiP-HA or EGFP-HA expressing T98G cells. Cells were treated with cycloheximide (CHX, 50 μg/mL) at indicated periods and analyzed by Western blot. EGFP-HA served as a control vector. HA antibody was used to show BiP-HA and EGFP-HA protein levels. β-Actin served as the loading control. **(D)** Ubiquitination assays for evaluating FKBP9 ubiquitination in T98G cells expressing FKBP9-HA infected with shCtr or shBiP-3#. The cells were treated with 5 μM MG-132 for 6 h and then lysed and subjected to immunoprecipitation assays using HA antibody. The FKBP9 ubiquitination was detected by Western blot with an anti-ubiquitin antibody. **(E)** Western blot analysis of FKBP9 protein in T98G cells expressing BiP-HA or EGFP-HA. Cells were treated with Tm (1 μg/mL) for the indicated times and analyzed by Western blotting.

**Figure S3:**
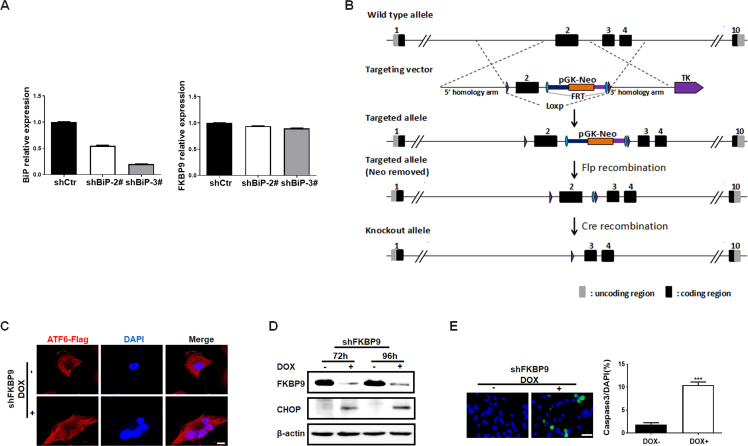
FKBP9 knockdown induces apoptosis by induction of lethal ER stress. **(A)** Quantitative PCR analysis of the relative RNA level of FKBP9 upon BiP knockdown by shRNAs of shBiP-2# and shBiP-3#. **(B)** The FKBP9 conditional allele (FKBP9 Flox) was created by inserting an Frt-flanked pGKNeoR cassette into intron 2 and the loxP sites at the 5ʹ end of exon 2 and intron 2. **(C)** Representative confocal microscopy images of ATF6-Flag translocation into the nucleus. T98G cells stably expressing tet-on shFKBP9 were infected with pTomo-ATF6-Flag lentivirus, and subjected to doxycycline-induced FKBP9 silencing to observe ATF6-Flag localization. ATF6-Flag and cell nuclei were stained with Cy3 and DAPI, respectively. DOX was used to induce FKBP9 silencing. Scale bar: 10 μm. **(D)** Western blotting was used to detect the expression changes in ER stress-related CHOP proteins for doxycycline-induced FKBP9 silencing at 72 h and 96 h in T98G cells. β-Actin was used as the loading control. **(E)** Immunofluorescent staining of cleaved-caspase3 in doxycycline-induced FKBP9-silenced T98G cells. Quantification data are shown at right. Data are presented as mean ± SD. ∗∗∗*P* < 0.001. Scale bar: 50 μm.

Given that BiP directly binds with FKBP9 in glioma cells, to determine the possible role of BiP in regulating FKBP9, we characterized the molecular consequences of BiP knockdown in glioma cells. We found that the protein level of FKBP9 was significantly decreased upon BiP knockdown (Fig. 3A) but had no significant effect on the mRNA level of the FKBP9 (Fig. S3A). To further support the results that FKBP9 was down-regulated upon BiP silencing and eliminated off-target effects of shRNA, we further constructed the HA-tagged BiP vector that was mutated in synonymous mutations to not targeted for degradation by shBiP-3#; the restoring BiP expression rescued FKBP9 protein expression in T98G compared with the control vector EGFP-HA (Fig. 3B). We hypothesized that BiP might regulate the stability of FKBP9 protein. We next used cycloheximide to inhibit protein synthesis for 6–24 h to analyze how FKBP9 was altered in T98G cells overexpressing HA-tagged BiP or EGFP. The results showed that BiP overexpression prolonged the half-life of the FKBP9 protein, and the relative level of FKBP9 protein was more stable in BiP-overexpressing cells than in control cells (Fig. 3C). A comprehensive examination of the outcomes obtained from both BiP silencing and overexpression could substantiate the role of BiP in upholding FKBP9 stability. Further experiments were performed to find that FKBP9 was ubiquitinated for degradation upon BiP silencing in T98G cells expressing FKBP9-HA (Fig. 3D). FKBP9 was reported to be ubiquitinated for degradation upon ER stress inducer treatment, and T98G cells overexpressing BiP or EGFP were treated with the ER stress inducer tunicamycin (Tm) for the indicated times. The results indicated that overexpression of BiP in T98G cells promoted FKBP9 stabilization compared with control cells upon Tm treatment (Fig. 3E). Taken together, these results indicate that BiP stabilizes the FKBP9 protein by directly interacting with it.

### BiP–FKBP9 axis correlates with poor survival in patients with glioma

**Figure 4:**
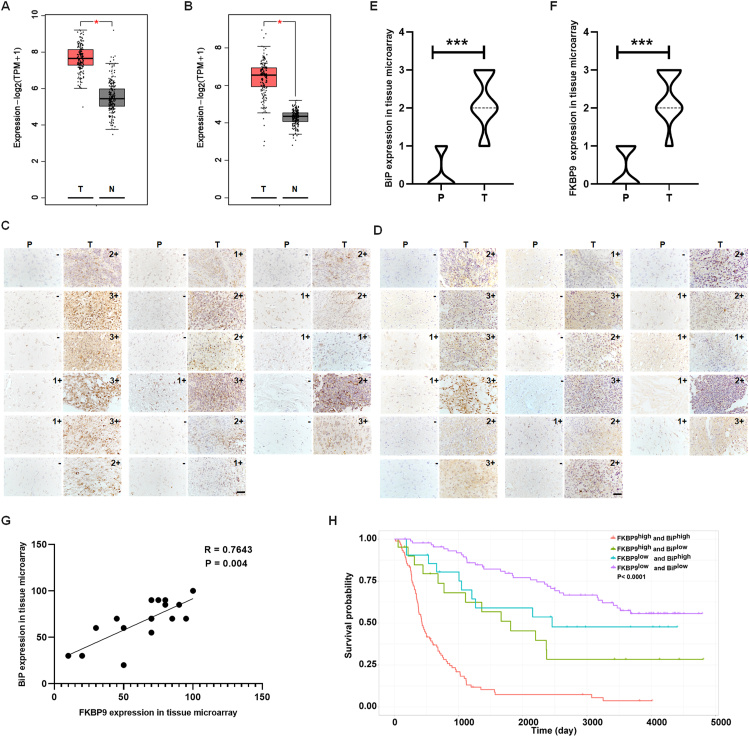
BiP–FKBP9 axis correlates with poor survival in patients with glioma. **(A)** The gene expression of BiP in glioma (red, *n* = 163) and normal (gray, *n* = 207) samples from TCGA and GTEx datasets. N: normal; T: tumor. ∗*P* < 0.05. **(B)** The gene expression of FKBP9 in glioma (red, *n* = 163) and normal (gray, *n* = 207) samples from TCGA and GTEx datasets. N: normal; T: tumor. ∗*P* < 0.05. **(C, D)** Immunohistochemical staining for BiP (C) and FKBP9 (D) expression in the tissue microarrays with glioma and corresponding para-tumor. P: para-tumor; T: tumor. The immunostaining was evaluated as “−” for minor staining, “1+” for mild staining, “2+” for moderate staining, and “3+” for strong staining. Scale bar, 50 μm. **(E, F)** Statistics of BiP (E) and FKBP9 (F) protein expression levels in para-tumor and tumor of the tissue microarray in (C, D). ∗∗∗*P* < 0.001. **(G)** Pearson correlation analysis for FKBP9 against BiP based on the immunohistochemical staining of the tissue microarray in (C, D). **(H)** Kaplan–Meier analysis of the overall survival probability of glioma patients based on FKBP9 and BiP expression status. According to the expression of FKBP9 and BiP, 222 patients were divided into FKBP9-high/BiP-high (*n* = 90), FKBP9-high/BiP-low (*n* = 21), FKBP9-low/BiP-high (*n* = 21), and FKBP9-low/BiP-low (*n* = 90) groups.

In order to examine the clinical significance of the BiP–FKBP9 axis in individuals diagnosed with glioma. We further confirmed that the mRNA and protein levels of FKBP9 and BiP were significantly up-regulated compared with normal tissue expression in the same database and samples, rather than understanding BiP and FKBP9 studied separately in previous studies. The mRNA expression of FKBP9 and BiP in glioma were compared with normal tissues in the TCGA and GTEx datasets^29^ (http://gepia2.cancer-pku.cn/#index). The results showed that BiP (Fig. 4A) or FKBP9 (Fig. 4B) expression in patients with glioma was significantly higher than that in normal tissues. To further validate the protein levels in tissue microarrays. Immunohistochemical analysis in a tissue microarray containing 17 adjacent normal brain tissues and glioma tissues showed that both BiP and FKBP9 expression were markedly elevated in tumor tissues compared with paired adjacent normal brain tissues (Fig. 4C–F). Importantly, the immunohistochemical expression analysis for FKBP9 plotted against BiP indicated a significant positive correlation between BiP and FKBP9 expression in patients with glioma (Fig. 4G). Further, the Chinese Glioma Genome Atlas (CGGA) database was utilized for Kaplan–Meier survival analysis,^30^ taking into account the expression levels of BiP and FKBP9. Consistent with previous studies, heightened FKBP9 expression significantly diminishes patient survival. As expected, the up-regulation of BiP exacerbated the adverse impact of FKBP9 on patient survival. The simultaneous high expression of two genes is associated with a worse prognosis, indicating that the BiP–FKBP9 axis, rather than individual expression, plays the most significant role in glioma (Fig. 4H).

### Ablation of Fkbp9 in glioma cells prolongs the survival of genetically engineered mice with orthotopic glioma by induction of lethal ER stress

**Figure 5:**
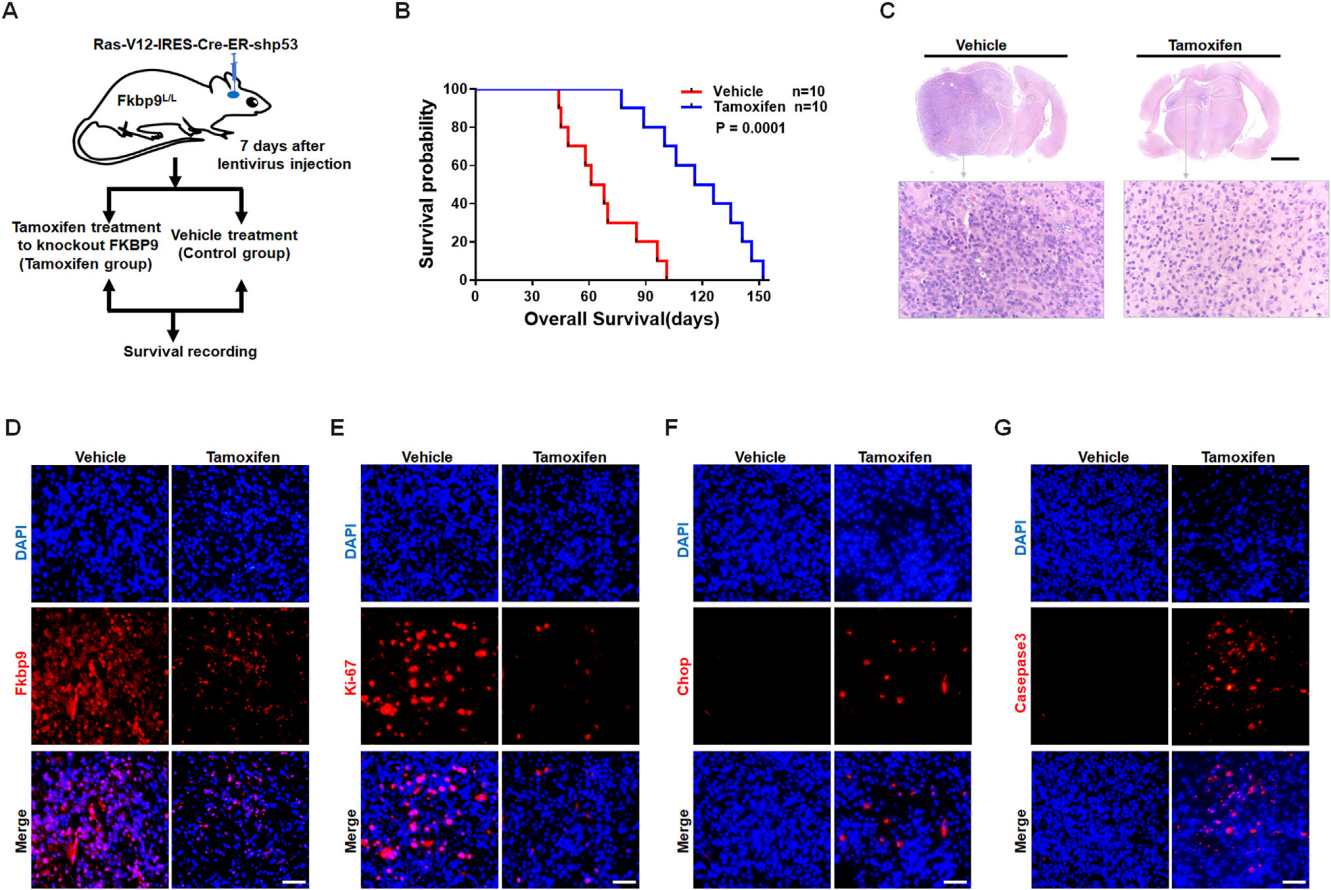
Ablation of FKBP9 in tumor cells prolongs the survival of genetically engineered mice with orthotopic glioma by induction of lethal ER stress. **(A)** Diagram of 8-week-old Fkbp9^L/L^ mice injected with Ras-V12-IRES-Cre-ER-shp53 lentivirus in the hippocampus. Tamoxifen (4 mg/20 g body weight) was administered to induce Fkbp9 knockout. The same volume of vehicle (oil) was administered in the control group. **(B)** Kaplan–Meier survival curves of Fkbp9^L/L^ mice who were injected intracranially with Ras-V12-IRES-Cre-ER-shp53 lentivirus and then administered tamoxifen (*n* = 10) or vehicle (*n* = 10). The points on the curves indicate deaths. *P* = 0.0001. **(C)** H&E staining of representative images in intracranial glioma tissues from the vehicle or tamoxifen group. The tissue in the dotted box indicates neoplastic tissue. The boxed tumor tissue is shown at higher magnification in the gross brain tumor. Scale bar: 2 mm. **(D‒G)** Immunofluorescence staining of glioblastoma from the control group and tamoxifen-treated group for Fkbp9 (D), Ki-67 (E), Chop (F), and cleaved-caspase3 (G). Scale bar: 50 μm.

Further to evaluate the effect of Fkbp9 knockout on the survival of immunocompetent mice with orthotopic glioblastoma, Fkbp9 conditional knockout mice (Fkbp9^L/L^) were generated as shown in Fig. S3B. Ras-V12-IRES-Cre-ER-shp53 lentivirus was injected into the hippocampus of Fkbp9^L/L^ mice to induce glioblastoma as previously described.^25^ Tamoxifen in oil was administered to delete Fkbp9 in infected cells 7 d after lentiviral infection (Fig. 5A). The results showed that Fkbp9 ablation significantly prolonged mouse survival (Fig. 5B) and H&E staining of gross brain specimens indicated less neoplastic tissue in the Fkbp9 knockout group (Fig. 5C). To investigate the mechanism by which Fkbp9 ablation prolongs overall survival of mice with glioblastoma. *In vitro* experiment showed that FKBP9 knockdown increased ATF6 translocation from the cytoplasm to the nucleus (Fig. S3C), and its silence also increased CHOP (Fig. S3D), a key player in ER stress-induced apoptosis,^31^ and activation of caspase3 apoptotic protein (Fig. S3E). As expected, immunohistochemical staining showed that tumors induced with tamoxifen had a smaller number of Fkbp9-positive cells (Fig. 5D) and a reduced proliferation rate as measured by Ki-67 (Fig. 5E) *in vivo*. Furthermore, tumors with Fkbp9 deletion showed an increase in the expression of Chop (Fig. 5F) and eventually activated cleaved-caspase3 to induce tumor cell apoptosis in orthotopic tumors (Fig. 5G). These results suggest that Fkbp9 knockout triggers ER stress-induced Chop-related apoptosis in tumor cells to prolong the survival of Fkbp9 genetically engineered mice with intracranial glioblastoma.

**Figure S4:**
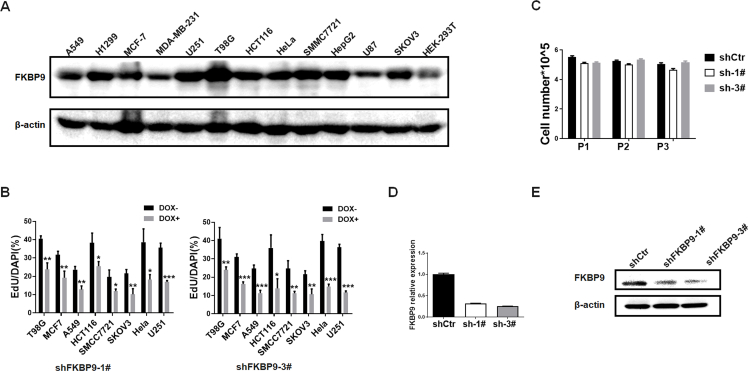
FKBP9 is essential for the survival of multiple cancer cell lines. **(A)** Western blots of FKBP9 protein levels in various cancer cell lines and HEK-293T cells. β-Actin was used as the loading control. **(B)** Quantification of positive EdU rates in shFKBP9-1# and shFKBP9-3# lentivirus-infected cancer cell lines. Data are presented as mean ± SD; ∗*P* < 0.05, ∗∗*P* < 0.01, ∗∗∗*P* < 0.001. **(C)** Cell count statistics of HEK-293T cells expressing pLKO.1-shCtr-puro, pLKO.1-shFKBP9-1#-puro, and pLKO.1-shFKBP9-3#-puro in three passages. Data are presented as mean ± SD. **(D, E)** Knockdown efficiency of FKBP9 mRNA-level (D) and protein level (E) in HEK-293T cells expressing shCtr, shFKBP9-1#, and sh-FKBP9-3# in (C) respectively.

We further identified that FKBP9 has relatively high levels in tumor cell lines including glioblastoma, breast cancer, lung cancer, and liver cancer, compared with non-transformed human cell line HEK-293T (Fig. S4A). Induced FKBP9 silencing inhibited cellular proliferation (Fig. S4B), as shown by EdU incorporation in MCF-7, A549, T98G, U251, HCT116, SMMC7721, HeLa, and SKOV3 cells. HEK-293T cells were infected with pLKO.1-shFKBP9-1#/3#-puro lentivirus to establish cell lines in which FKBP9 was constitutively silenced. These cells were serially passaged thrice, and the cell number was counted at every passage. The results showed that FKBP9 knockdown did not result in any significant change in cell number even after three passages in HEK-293T cells (Fig. S4C), although the knockdown efficacy was confirmed at both the mRNA and protein levels, as shown in Figure S4D and E. These data suggest that FKBP9 is more essential for the survival of cancer cells than for non-cancer cells, implying that FKBP9 may be a potential target for cancers with few side effects.

## Discussion

FKBP9 has been reported to play important roles in glioma, but the factors that regulate FKBP9 expression have never been investigated. In this study, co-immunoprecipitation and mass spectrometry analysis indicate that the chaperone BiP interacts with FKBP9, and SPR analysis with purified BiP and FKBP9 proteins further confirmed that BiP directly bound to FKBP9 with high affinity. FKBP family members were previously reported to interact with BiP through different domains; for example, FKBP22 in *Neurospora crassa* binds to BiP through the FKBP domain,^32^ and binding between mouse FKBP23 and BiP is mediated by the EF-hands domain in a Ca^2+^-dependent manner.^33^ Human FKBP10, the closest relative of FKBP9, was found in a complex with HSP47, BiP, and LH2 in the ER and may regulate the telopeptide lysyl hydroxylation of type I procollagen.^34^ Whether FKBP10 directly interacts with BiP remains unknown. Both FKBP9 and FKBP10 contain four PPIase domains at the amino-terminal and two EF-hand domains at the carboxy-terminal. Truncated FKBP9 without carboxy-terminal EF-hands that can efficiently coprecipitate with BiP demonstrated that EF-hands are not necessary for binding. Further deletion of two C-terminal PPIase domains did not interfere with its co-existence in the complex, indicating that the two residual N-terminal PPIase domains may be responsible for the binding. The roles and mechanisms of BiP binding by different domains remain to be further elucidated.

BiP plays crucial roles in ER homeostasis and is a central sensor for ER stress.^35^ Tumor progression requires BiP for cancer cell survival, angiogenesis, metastasis, and resistance to therapy.^36^^,^^37^ It is a core chaperone in promoting protein folding, assembly, and transport in the ER, as well as the removal of misfolded proteins through ER-associated protein degradation.^38^ BiP functions crucially depend on many interaction partners, including co-chaperones, nucleotide exchange factors, and signaling molecules.^39^ BiP has been reported to stabilize various oncoproteins, such as programmed death-ligand 1(PD-L1),^40^ clusterin (CLU),^41^ and CHOP.^42^ Our results show that BiP promotes FKBP9 protein stabilization and patients with both high expression of BiP and FKBP9 show a worse prognosis, indicating that the BiP–FKBP9 axis may play an important role in glioma patients. Further mechanistic studies and large-scale screening of patient samples are deserved to determine whether targeting the BiP–FKBP9 axis is a potentially effective therapeutic strategy for multiple types of cancers.

FKBP9 is an ER-resident protein that serves as a molecular chaperone in glioblastoma, aiding protein folding and mitigating protein misfolding in the ER lumen.^21^ Our research, in conjunction with previous study,^21^ has shown that the down-regulation of FKBP9 leads to an accumulation of misfolded proteins, thereby inducing ER stress and subsequently triggering apoptosis mediated by CHOP. The silencing of FKBP9 could potentially initiate CHOP-linked apoptosis by disrupting the balance of ER stress response. As we noted, the depletion of FKBP9 may indeed release BiP, another critical ER chaperone involved in protein folding. However, BiP is abundantly and excessively expressed in cells,^43^^,^^44^ exceeding FKBP9 expression by more than 100-fold in glioma cells.^45^ As such, the knockdown of FKBP9 is theoretically expected to have a minimal impact on the abundance of free BiP. Furthermore, our study revealed that BiP and FKBP9 form a complex, and this interaction can stabilize FKBP9. This suggests that the function of the BiP and FKBP9 complex is not entirely redundant, and they may have distinct roles in managing protein folding in the ER. More detailed investigations are required to further elucidate the specific roles of BiP and FKBP9 in the ER stress response and the potential crosstalk between these two proteins.

We further expanded that the knockdown of FKBP9 inhibited the proliferation of various tumor cell lines. Conversely, loss of FKBP9 expression does not induce severe consequences as shown by the following evidence: i) FKBP9 silencing had no significant effects on cell death and proliferation in the non-malignant human cell line HEK-293T; ii) mice with the constitutive deletion of the Fkbp9 gene generally develop and reproduce normally; iii) another Fkbp9 gene knockout mouse strain generated by EUCOMM (IMPC, International Mouse Phenotyping Consortium, MGI: 1350921) also showed that Fkbp9 ablation is not fatal and develops normally except for phenotypes in homeostasis/metabolism or adipose tissue, such as decreased lactate dehydrogenase levels, circulating total protein levels, circulating aspartate and alanine transaminase levels, and circulating sodium and chloride levels. These data suggest that FKBP9 may be a potential therapeutic target for multiple types of cancer with minimal side effects.

## Ethics declaration

The approval for this study was granted by the animal ethics committee of Kunming Institute of Zoology, Chinese Academy of Sciences (SMKX-20170102-01).

## Author contributions

S.R.L., W.X.X., and B.S. designed and performed the experiments. S.R.L and X.D.Z. wrote the main manuscript. W.X.X provided insights and analyzed the data. H.Y. and S.H. analyzed the data. J.G. and H.Z. performed LC‒MS/MS data analysis. W.Y.P., D.Y. and Y.J.Z. provided support with the experiments. X.D.Z., Q.P.K., and T.X.X contributed to the interpretation of the data and supervised the study. All authors read and approved the final manuscript.

## Conflict of interests

The authors declare that there are no competing interests.

## Funding

This work was supported by the 10.13039/501100001809National Natural Science Foundation of China (No. 82103107 to B.S.), the open project from the State Key Laboratory of Genetic Resources and Evolution of China (No. GREKF19-06 to H.Y.), and the 1.3.5 project for disciplines of excellence, West China Hospital, Sichuan University (No. ZYYC20002 to X.D.Z.).
